# Dynamic Brain Interactions during Picture Naming

**DOI:** 10.1523/ENEURO.0472-18.2019

**Published:** 2019-07-03

**Authors:** Aram Giahi Saravani, Kiefer J. Forseth, Nitin Tandon, Xaq Pitkow

**Affiliations:** 1 Department of Neuroscience, Baylor College of Medicine, One Baylor Plaza, Houston, TX 77030; 2 Department of Neurosurgery, University of Texas Health Science Center, 6431 Fannin Street, Houston, TX 77030; 3 Mischer Neuroscience Institute, Memorial Hermann Hospital Texas Medical Center, Houston, TX 77030; 4 Department of Electrical and Computer Engineering, Rice University, 6100 Main St., Houston, TX 77005; 5 Center for Neuroscience and Artificial Intelligence, Baylor College of Medicine, One Baylor Plaza, Houston, TX 77030

**Keywords:** dynamics, electrocorticography, Hidden Markov Model, language, network

## Abstract

Brain computations involve multiple processes by which sensory information is encoded and transformed to drive behavior. These computations are thought to be mediated by dynamic interactions between populations of neurons. Here, we demonstrate that human brains exhibit a reliable sequence of neural interactions during speech production. We use an autoregressive Hidden Markov Model (ARHMM) to identify dynamical network states exhibited by electrocorticographic signals recorded from human neurosurgical patients. Our method resolves dynamic latent network states on a trial-by-trial basis. We characterize individual network states according to the patterns of directional information flow between cortical regions of interest. These network states occur consistently and in a specific, interpretable sequence across trials and subjects: the data support the hypothesis of a fixed-length visual processing state, followed by a variable-length language state, and then by a terminal articulation state. This empirical evidence validates classical psycholinguistic theories that have posited such intermediate states during speaking. It further reveals these state dynamics are not localized to one brain area or one sequence of areas, but are instead a network phenomenon.

## Significance Statement

Cued speech production engages a distributed set of brain regions that must interact with each other to perform this behavior rapidly and precisely. To characterize the spatiotemporal properties of the networks engaged in picture naming, we recorded from electrodes placed directly on the brain surfaces of patients with epilepsy being evaluated for surgical resection. We used a flexible statistical model applied to broadband gamma to characterize changing brain interactions. Unlike conventional models, ours can identify changes on individual trials that correlate with behavior. Our results reveal that interactions between brain regions are consistent across trials. This flexible statistical model provides a useful platform for quantifying brain dynamics during cognitive processes.

## Introduction

Neural computation requires the orchestration of distributed cortical processes. In many complex cognitive tasks, it is unlikely that interactions between large populations of neurons merely generate a simple feedforward sequence of activated brain regions. Instead, these processes likely involve distributed interactions that change over time and depend on context. To understand information flow and computation in the brain, it is critical to account for these dynamic interactions between functionally distinct regions.

For simplicity, most analyses of coarse-grained brain activity like that measured by electrocorticography (ECoG) are based on assumptions of linearity. These include methods based on second-order correlations (Aertsen et al., 1987; Friston et al., 1993), structural equation modeling (Horwitz, 1994; Penny et al., 2004), Granger causality (Granger, 1969; Baccalá and Sameshima, 2001; Josic et al., 2009), Gaussian graphical models (Zheng and Rajapakse, 2006; Rajapakse and Zhou, 2007), and linear dynamical systems. Such linear models cannot capture crucial behaviors like flexible time-dependent or context-dependent interactions. One way to improve the expressiveness of these models while preserving some of their tractability is to use switching linear dynamics, where the switch determines which linear dynamical system currently best describes the neural dynamics.

Here, we present the first application of such a dynamical model to direct recordings from human brains during language production. We applied an autoregressive (AR) hidden Markov model (HMM) or ARHMM, a type of hierarchical Bayesian network, that accounts for the observed continuous time series as a consequence of switching between a discrete set of network states that govern the electrical activity. Each discrete state corresponds to distinct stochastic linear dynamics for the observed recordings. These switching dynamics between the different network states approximates the nonlinear dynamics of the full system. Our statistical method learns these state dynamics and the latent state transition matrix, as well as the trial-specific state sequences (see below, ARHMM).

Hidden Markov Models (HMMs) have become a useful tool in characterizing brain dynamics on a multitude of spatial and temporal scales. They have proved valuable in brain-computer interfaces (Kemere et al., 2008; Al-Ani and Trad, 2010), modeling the sequential structure of cognitive processes using functional magnetic resonance imaging (Anderson, 2012), and for describing brain states and behavioral states (Abeles et al., 1995; Sahani, 1999a; Escola et al., 2011). To capture not only states but also dynamics, recent studies of human electrocorticography (ECoG) data have used ARHMMs to classify dynamical modes of epileptic activity (Wulsin et al., 2014; Baldassano et al., 2016), a neural state characterized by relatively simple dynamics in comparison to healthy brain function.

In the present work, we use ARHMMs with high-resolution intracranial human electrophysiology to classify dynamical states and to reveal information flow between brain areas during normal cognition. We analyze neural activity evoked during picture naming, an essential language task that requires several interlocking cognitive processes. This uniquely human ability requires visual processing, lexical semantic activation and selection, phonological encoding, and articulation. We concentrate our analysis on broadband γ power, a neural measure that is thought to arise from spike surges, and serves as a measure of local cognitive processing (Logothetis, 2003; Lachaux et al., 2012). We demonstrate that ARHMMs can identify and characterize transient network states in ECoG data during a language task.

Picture naming is one often studied language task that has been the backbone of many psycholinguistic theories of speech production. Chronometric studies of picture naming (Levelt, 1989), as well as the nature of common speech errors (Fromkin, 1971) and speech disruption patterns in aphasia (Dell et al., 1997a), have led to theories that linguistic components are organized hierarchically and assembled sequentially. Yet there are no data that can directly be used to validate these ideas, and the dynamics of the cortical networks supporting even simple, single-word articulations, remain unknown. In the absence of such data, it is difficult to resolve competing models such as between discrete (Fromkin, 1971; Garrett, 1980; Indefrey and Levelt, 2004) and interactive (Dell et al., 1997b; Rapp and Goldrick, 2000) models of language production.

Prior studies have leveraged the high spatiotemporal resolution of intracranial electroencephalography to study specific brain regions during language production (Sahin et al., 2009; Edwards et al., 2010; Conner et al., 2014; Kadipasaoglu et al., 2016; Forseth et al., 2018), and have applied adaptive multivariate AR (AMVAR) analysis (Ding et al., 2000; Korzeniewska et al., 2008; Whaley et al., 2016), to reveal the fast, transient dynamics of human cortical networks. This class of methods assumes a consistent progression of state sequences across trials, an assumption that is frequently violated during complex behaviors like language that invoke multiple cognitive processes. Consequently, the inferred activity patterns from AMVAR may be grouped into false network states. The ARHMM analysis developed here is a principled probabilistic framework to resolve trial-by-trial network state dynamics. As an added benefit, it also provides model uncertainties. We demonstrate that this method delivers improved estimates of network dynamics in human language function compared to conventional AMVAR clustering analyses.

We show that unsupervised Bayesian methods can infer reliable time series of latent network states and information flow from ECoG signals during a task requiring integration of visual, semantic, phonological, and sensorimotor processing. These states have dynamics that are consistent across subjects and reflect the timing of subjects’ actions. From the trial-resolved sequence network states we learn additional characteristics of the network states not available through fixed-timing models like AMVAR.

## Materials and Methods

### Human subjects

We enrolled three patients (one male, two female; mean age 28 ± 9 years; mean IQ 86 ± 3) undergoing evaluation of intractable epilepsy with subdural grid electrodes (left, *n* = 2; right, *n* = 1) in this study after obtaining informed consent. Human subjects were patients undergoing intracranial evaluation at the Texas Comprehensive Epilepsy program at Memorial Hermann Hospital, in accordance with a study protocol approved by the institutional committee on the protection of human subjects. Hemispheric language dominance was evaluated by intracarotid sodium amytal injection (Wada and Rasmussen, 2007), fMRI laterality index (Ellmore et al., 2010; Conner et al., 2011), or cortical stimulation mapping (Tandon, 2008; Forseth et al., 2018). All patients were found to have left-hemisphere language dominance.

### Experimental design

**Figure 1.**
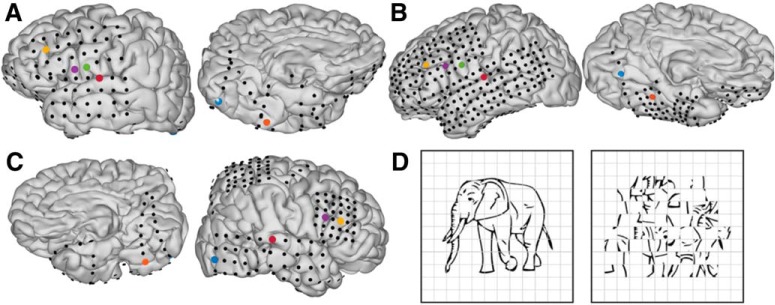
***A–C***, Individual pial surface and electrode reconstructions. One representative electrode was selected from each of the following regions: early visual cortex (blue), middle fusiform gyrus (orange), pars triangularis (yellow), pars opercularis (purple), ventral sensorimotor cortex (green), and auditory cortex (red). ***D***, Picture naming stimuli: coherent (left) and scrambled (right).

Subjects engaged in a visual naming task (Fig. 1*D*, left). We instructed subjects to articulate the name for common objects depicted by line drawings (Snodgrass and Vanderwart, 1980; Kaplan et al., 1983). Subjects were instructed to report “scrambled” for control images in which we randomly rotated pixel blocks demarcated by an overlaid grid (Fig. 1*D*, right). Each visual stimulus was displayed on a 15-inch LCD screen positioned at eye level for 2 s with an interstimulus interval of 3 s. A minimum of 240 images and 60 scrambled stimuli were presented to each patient using presentation software (Python v2.7).

### MR acquisition

Preoperative anatomic MRI scans were obtained using a 3T whole-body MR scanner (Philips Medical Systems) fitted with a 16-channel SENSE head coil. Images were collected using a magnetization-prepared 180° radio frequency pulse and rapid gradient-echo sequence with 1 mm sagittal slices and an in-plane resolution of 0.938 × 0.938 mm (Conner et al., 2011). Pial surface reconstructions were computed with FreeSurfer (v5.1; Dale et al., 1999) and imported to AFNI (Cox, 1996). Postoperative CT scans were registered to the preoperative MRI scans to localize electrodes relative to cortex. Subdural electrode coordinates were determined by a recursive grid partitioning technique and then validated using intraoperative photographs (Pieters et al., 2013).

### ECoG acquisition

Grid electrodes (*n* = 615), subdural platinum-iridium electrodes embedded in a SILASTIC sheet (PMT Corporation; top-hat design; 3-mm diameter cortical contact), were surgically implanted via a craniotomy (Tandon, 2008; Conner et al., 2011; Pieters et al., 2013). ECoG recordings were performed at least 2 d after the craniotomy to allow for recovery from the anesthesia and narcotic medications. These data were collected at either a 1000- or 2000-Hz sampling rate and a 0.1- to 300- or 0.1- to 700-Hz bandwidth, respectively, using NeuroPort NSP (Blackrock Microsystems). Continuous audio recordings of each patient were made with an omnidirectional microphone (3- to 20,000-Hz response, 73-dB signal-to-noise ratio (SNR), Audio Technica U841A) placed next to the presentation laptop. These were analyzed offline to transcribe patient responses and to determine the time of articulation onset and offset (Forseth et al., 2018).

### Data processing

From previous work, we identified six anatomic regions of interest that broadly span the cortical network engaged during picture naming (Conner et al., 2014; Forseth et al., 2018): early visual cortex, mid-fusiform gyrus, pars triangularis, pars opercularis, ventral sensorimotor cortex, and superior temporal gyrus. In each individual, we selected the most active electrodes from each region (Fig. 1*A*,*B*). In a separate analysis to combine information from multiple sources, we grouped electrodes from each brain region, computed the principal components of the high γ-band power across electrodes within each region and selected the leading component as a meta-electrode to represent each region. Electrodes used for analysis were uncontaminated by epileptic activity, artifacts, or electrical noise. Furthermore, we analyzed only trials in which cortical activity did not show evidence of epileptiform artifact (Conner et al., 2014; Kadipasaoglu et al., 2014).

We selected trials with reaction times >600 and <2800 ms. Data were re-referenced to a common average of electrodes without epileptiform activity. The analytic signal was generated by frequency-domain bandpass Hilbert filters featuring paired sigmoid flanks with half-width of 1 Hz (Forseth et al., 2018). Instantaneous power was then extracted as the squared magnitude of the analytic signal, normalized by a prestimulus baseline level (700–200 ms before picture presentation), and then downsampled to 200 Hz.

To initialize the ARHMM, we estimated the dynamics in 100-ms time windows with 50-ms overlap, using the AMVAR estimation method of (Ding et al., 2000). The AMVAR estimates were clustered (*k*-means clustering with Euclidean distance norm) into a set of discrete states to initialize the ARHMM inference.

### ARHMM

AR processes are random processes with temporal structure, where the current state ***x_t_*** of a system is a linear combination of previous states and a stochastic innovation ***v****_t_* ∼ *N*(0, *I* ) (zero mean isotropic white noise). The dynamics of such a system is thus both linear and stochastic. This stochastic linear dynamics can be described by a tensor of AR coefficients, ***A*** = {*A_τ_*} (i.e., a matrix for each time lag *τ*), and a covariance matrix *Q* for the stochastic aspect of the system:

(1) $$x_{t}=\overset{N_{\tau }}{\underset{\tau =1}{\sum }}A_{\tau }{\;x}_{t-\tau }+Q^{1/2}v_{t},$$

where *N_τ_* is the order (number of relevant time lags) of the AR process. Since this model is linear, it is poorly suited to describing brain activity, which motivates us to use a richer model with changing dynamics.

A HMM is a latent state model which describes observations as a consequence of unobserved discrete states *z*, where each state emits observable variables with specific probabilities. The probability of occurrence of a state depends on the previous state, the defining characteristic of a Markov model. The set of transition probabilities constitute the state transition matrix,

(2) $$\Phi_{zz'}=P\left(z'|z\right)$$

where *z*′ and *z* are the current and previous state, respectively.

**Figure 2.**
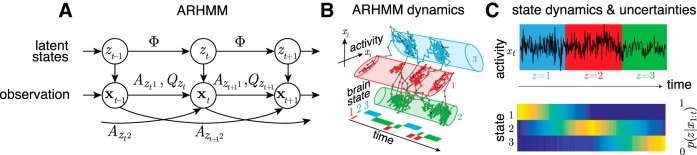
***A***, Graphical representation of an ARHMM with autoregressive (AR) order 2: latent states *z* and observations *x* evolve according to state transition matrix Φ, AR coefficients ***A***, and process covariance *Q*. ***B***, Illustration of the ARHMM latent state space model. ***C***, Simulated time series of data points emitted by three latent states (top) with inferred state probabilities (bottom).

The ARHMM combines AR stochastic linear dynamics with the HMM (Ghahramani and Hinton, 2000; Fox et al., 2009): Each latent state *z* indicates a different stochastic linear process with state-specific dynamics and process noise covariance, ***A****_z_* and *Q_z_* (Fig. 2*A*,*B*). The switching of the linear dynamics makes the ARHMM effectively nonlinear.

Applied to the multivariate ECoG data, each state describes the measured neural activity by a specific linear and stochastic dynamic, with a set of AR coefficients, *A*_***z***τ***ij***_, which specify the (Granger) causal dynamical relationship between nodes *i* and *j* for state *z* at time lag *τ*. For a given state *z* at time *t*, the multivariate autoregressive (MVAR) coefficients constitute an MVAR tensor, *A*_***ztτ***_, describing the evolution of the multivariate ECoG signal ***x*** at time *t*,

(3) $$x_{t}=\overset{N_{\tau }}{\underset{\tau =1}{\sum }}A_{z_{t}\tau }{\;x}_{t-\tau }+Q_{z_{t}}^{1/2}v_{t}+\mu_{z_{t}},$$

where ***v*** ∼ *N*(0, *I*) and ***μ****_z_* is a state-dependent bias.

Neither the set of dynamical parameters, {***A***, *Q*, ***μ***}, nor the occurrence and frequency of individual states (represented by the state transition matrix Φ), are observed. The ARHMM infers the latent parameters: the time series of network states *z_t_*, their transition probabilities Φ*_zz_*_′_, and the dynamical parameters for each state, ***A****_z_*, *Q_z_*, and ***μ***_*z*_.

This is in contrast to the commonly used method in ECoG signal analysis to estimate the autoregression coefficient matrix ***A*** and process noise covariance, *Q*, based on the assumption that the network states occur at same times across all trials (Morf et al., 1978; Ding et al., 2000). This assumption will inevitably be inaccurate when there are significant variations in the emergence and durations of state sequences. This leads to artifacts in the estimation of the state parameters and the creation of pseudo-states that combine data points from different states into a new mixed state estimate. Also, our method does not require any manual alignment of trials by the epoch of interest, such as stimulus onset or articulation onset (Whaley et al., 2016), but instead provides a means of predicting these events from brain activity.

Probabilistic inference alleviates this problem by assigning posterior probabilities (conventionally called “responsibilities”), *P*(*z_t_*|***x***_1:_*_T_*), to each state *z_t_* given the entire observed data sequence ***x***_1:_*_T_* (Fig. 2*C*,*D*). Since neither responsibilities nor dynamical parameters are known a priori, estimates for state parameters and responsibilities are calculated iteratively by an expectation-maximization algorithm (EM; Dempster et al., 1977) known as the Baum–Welch algorithm (Baum et al., 1970). We incorporate a prior over Φ to favor infrequent transitions, by adding pseudocounts of self-transitions to the observed state transitions. This is realized by adding a scaled identity matrix, *I*, to Φ, and then renormalizing:

(4) $$\Phi_{\text{smooth}}=\frac{\Phi +uI}{1+u}$$

where *u* sets the lower bound on the time constant of self-transitions, and thereby determine minimal average state durations. For the state transition matrix (Eq. 2), we used a flat prior on transitions between different states, and a “stickiness” parameter, *u* = 0.5, for transitions back to the same state.

Initial conditions for ***A*** and *Q* are informed by the lagged correlation MVAR clustering method from (Morf et al., 1978; Ding et al., 2000). We initialized the state-dependent biases ***μ****_z_* with random seeds. For the expectation part of our algorithm, responsibilities within each trial are evaluated based on the previous iteration’s parameters from the maximization loop. The maximization in turn uses these responsibilities to attribute the data points to different network states when estimating new parameters. The number of states and time lags in the ARHMM is selected according to the Bayesian information criterion (BIC; Schwarz, 1978).

### Visualizing network states

The ARHMM classifies dynamical states by the network connectivity associated with the inferred MVAR coefficient matrix. The MVAR coefficients and the related partial directed coherence (PDC) in the frequency domain are measures of causality for interactions between brain areas. PDC was defined by Baccalá and Sameshima (2001) to describe information flow (in the sense of Granger causality) between multivariate time series in the frequency domain. This measure is directly related to the MVAR coefficients, and for each state, *z*, we have:

(5) $$\pi_{zjk}\left(f\right)=\frac{{\overset{¯}{A}}_{zjk}\left(f\right)}{\left\|{\overset{¯}{A}}_{zk}\left(f\right)\right\|},$$

where

(6) $${\overset{¯}{A}}_{zjk}\left(f\right)=\{\begin{array}{cc} 1-\overset{N_{\tau }}{\underset{\tau =1}{\sum }}A_{z\tau jk}e^{-2\pi if\tau } & \text{for }j=k\\ -\overset{N_{\tau }}{\underset{\tau =1}{\sum }}A_{z\tau jk}e^{-2\pi if\tau }, & \text{otherwise.} \end{array}$$

represents the transfer function at frequency *f*, $\left\|{\overset{¯}{A}}_{zk}\left(f\right)\right\|={\left(\underset{j}{\sum }{\overset{¯}{A}}_{zjk}^{*}\left(f\right){\overset{¯}{A}}_{zjk}\left(f\right)\right)}^{1/2}$ is the norm of the *k*th column of ${\bar{A}}_{z}\left(f\right)$, and ^*^ denotes the complex conjugate. PDC is normalized to show the ratio between the outflow from channel *k* to channel *j* to the overall outflow from channel *k*, with $\left|\pi_{zjk}\right|$ taking values from the interval [0,1].

For each latent state *z*, we have an associated set of MVAR coefficients ***A****_z_*. We display these as directed graphs, with interactions quantified by the PDC magnitude $\left|\pi_{zjk}\right|$ integrated over all frequencies. In these graphs, each node represents a single electrode and arrows represent the causal relationship between nodes.

To visualize the inferred time series of network states with their associated probability we display the statistically most likely sequence of states (Viterbi trace) weighted by the associated uncertainty (responsibility).

To estimate the cumulative durations of each state, we first filter the states by computing the most probable state within a sliding 200-ms time window. We then integrate the total time within these filtered state sequences when each state dominated, from stimulus onset to 3200 ms after stimulus onset. We identify the end of the “language processing” state as the last time between picture presentation and articulation completion when the majority of states within a sliding 100-ms window from stimulus onset to articulation offset were identified as language processing.

### Robustness of ARHMM inference

**Figure 3.**
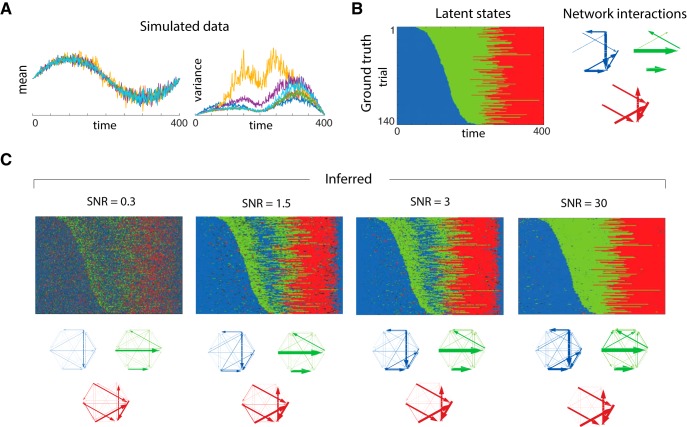
Comparison of state clustering for simulated data. ***A***, Underlying data with varying state durations, and mean-dependent variance with associated state sequence and network states on the right. ***B***, Ground truth: rasters of discrete latent states over time and trials and graphs of network interactions for each latent state, measured by PDC. ***C***, Estimates inferred from data with four different SNRs (0.3, 1.5, 3, and 30). Estimates are obtained from mean-subtracted time series. Color represents the discrete states, and confidence (responsibility) is encoded by brightness and increases with SNR.

To see how robust the ARHMM is to deviations from our model assumptions, we simulated data with mean-dependent process noise variance and observation noise. Note that uncontrolled or unmodeled factors can have similar effects as observation noise, creating unexplained variability. Figure 3*A* shows the simulated multivariate signal and its mean-dependent variance. Initialized from AMVAR-based *k*-means clustering of network states, the ARHMM then infers the trial-by-trial state sequence and network interactions for each state (Fig. 3*C*). Depending on the amount of observation noise [signal-to-noise ratios (SNR) = 0.3, 1.5, 3, and 30], the inference has different degrees of confidence, visualized by the brightness. The inferred states and network properties in each state generally agree with the ground truth (Fig. 3*B*) despite the model mismatch from observation noise and mean-dependent variance. The beginning and the end of the trials have similar properties, and for lower SNRs (more observation noise) the ARHMM naturally identifies them with the same latent state. Interestingly, in these conditions the ARHMM also misclassifies states in the middle of the trials, as it automatically finds hallmarks of the first state in the middle of the trial. For smaller observation noise, these states are correctly classified. Even when some times are misclassified in low SNR, the network structures are recovered accurately (Fig. 3*C*).

To estimate how well our model explains the temporal structure of the data we follow (Ding et al., 2000) and perform a residual whiteness test for our model fit, computing the auto-correlation and cross-correlation of the multivariate residuals between the ARHMM fit and the data for each time lag (except zero) up to AR model order. If the model captures the temporal structure of the data, the auto- and cross-correlation coefficients of the residuals should approach zero (uncorrelated white noise). For the SNRs tested, the residual temporal correlations between data and model fit were small, indicating that the model is reasonable even for low SNR.

### Statistical analysis

Correlations are calculated using the Pearson correlation. Significance was evaluated using a two-sided test for deviations from zero correlation. This was evaluated using the Fisher transform, which renders correlations approximately normal and thereby gives *p* values as $p=1+\text{erf}\left(-\frac{1}{\sqrt{2}}\text{arctanh}\left(\left|r\right|\right)\sqrt{n-3}\right)$ where *n* is the number of samples and *r* is the measured correlation coefficient (Fisher, 2006).

To compare the states across subjects, we define a dissimilarity score based on a distance, *d* (*A*, *B*), between two matrices of integrated PDC magnitudes:

(7) $$d\left(A,B\right)\equiv {\left\|\frac{A}{{\left\|A\right\|}_{F}}-\frac{B}{{\left\|B\right\|}_{F}}\right\|}_{F}$$

where ${\left\|A\right\|}_{F}=\sqrt{\underset{j,k}{\sum }{\left|A_{jk}\right|}^{2}}$ denotes the Frobenius norm. We calculate this difference for all pairs of states for different subjects.

### Code accessibility

The code/software described in the paper is freely available online at https://github.com/agiahi/ARHMM.git. The code is available as Extended Data 1.

10.1523/ENEURO.0472-18.2019.ed1 Extended Data 1 Code for the ARHMM. Download Extended Data 1, ZIP file.

### Computer system

The ARHMM code was run in MATLAB 2016b, on a MacBook Pro 13-inch 2016 computer system (2 GHz Intel Core i5, macOS 10.13.3).

## Results

**Figure 4.**
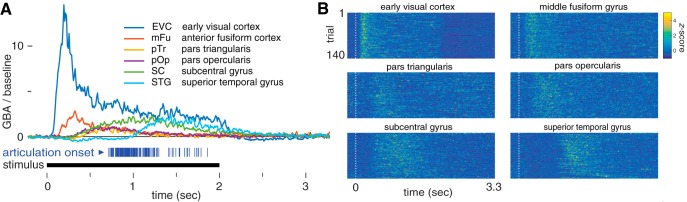
GBA at salient brain regions following picture presentation. ***A***, Trial-averaged GBA, relative to baseline. Each articulation onset is indicated by a vertical blue line below (mean 1.2 s), and the visual stimulus is presented during the black interval. ***B***, Density plot of trial-wise GBA, *z*-scored.

We observed a sequence of peak trial-averaged gamma-band activity (GBA; Fig. 4*A*) beginning in early visual cortex, moving anteriorly to middle fusiform gyrus and pars triangularis, and then culminating in pars opercularis, subcentral gyrus, and superior temporal gyrus. Consistent with Conner et al. (2014), Figure 4*B* indicates that the GBA response varies substantially across trials. This motivates an analysis method like the ARHMM that can account for the such variability.

**Figure 5.**
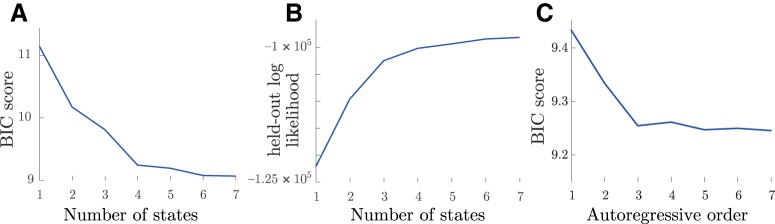
Model selection. BIC (***A***) and held-out log-likelihood (***B***) as a function of number of states. ***C***, BIC as a function of AR model order (number of time delays).

**Figure 6.**
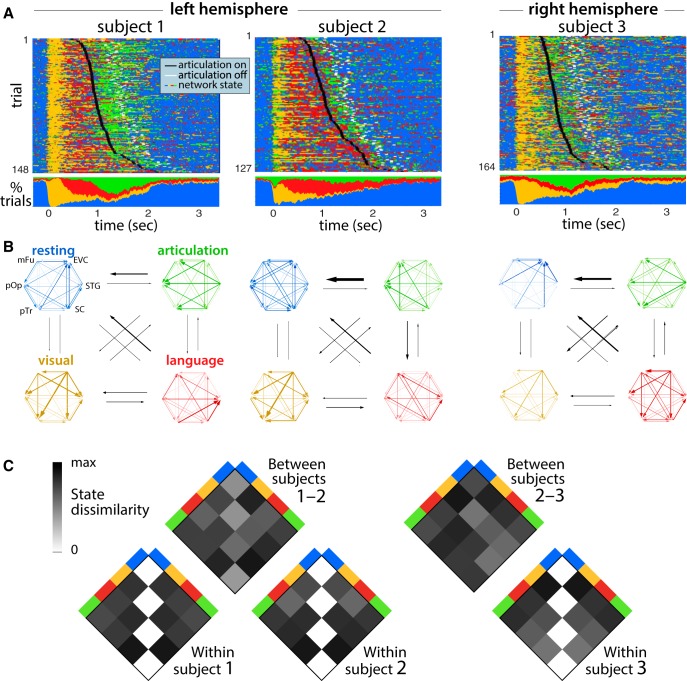
State sequences in brain activity. ***A***, Time dependence of most probable states from left and right hemispheric brain activity for three subjects. For each of six brain regions, we selected the most active electrode, fit the ARHMM to the broadband γ power on these six electrodes, and then estimated the sequence of latent brain states that best explains the observed activity. The top shows states as a trial-by-trial raster, and the bottom shows the fraction of trials on which each state was most probable. ***B***, Interactions between brain regions during the corresponding named network states, plotted as in Figure 3*C*. Black arrows indicate state transition probabilities according to the inferred state transition matrix. ***C***, Dissimilarity between brain states between and within subjects. Dissimilarity is measured as the difference between integrated PDC magnitude, according to Equation 7. Extended Data Figure 6-1 shows the same analysis, using not the most active electrodes in each region but instead the multi-electrode activity patterns with greatest variance within each region.

10.1523/ENEURO.0472-18.2019.f6-1 Extended Data Figure 6-1 State sequences in brain activity, plotted as in Figure 6, but for meta-electrodes created by selecting the principal component for subsets of electrodes in each brain region. States estimated from meta-electrodes produced crisper states (***A***) but were less similar across subjects (***B***, ***C***) than observed when selecting single electrodes with strongest signals (Fig. 6). Download Figure 6-1, EPS file.

**Figure 7.**
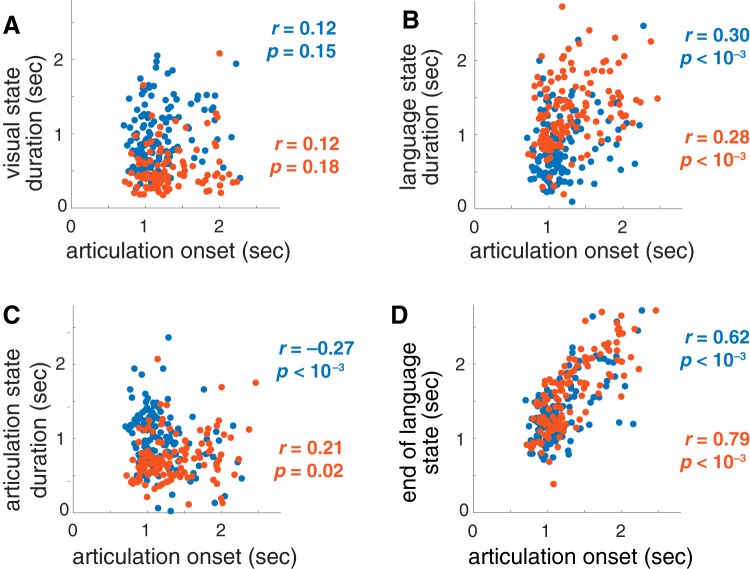
Duration of the active states; (***A***) visual processing, (***B***) language processing, and (***C***) articulation, as well as (***D***) the termination of the “language” state, compared to reaction times. Two patients’ left hemispheres (blue and orange) are plotted. The Pearson correlation coefficient *r* and *p* value for the null hypothesis of uncorrelated values are shown.

**Figure 8.**
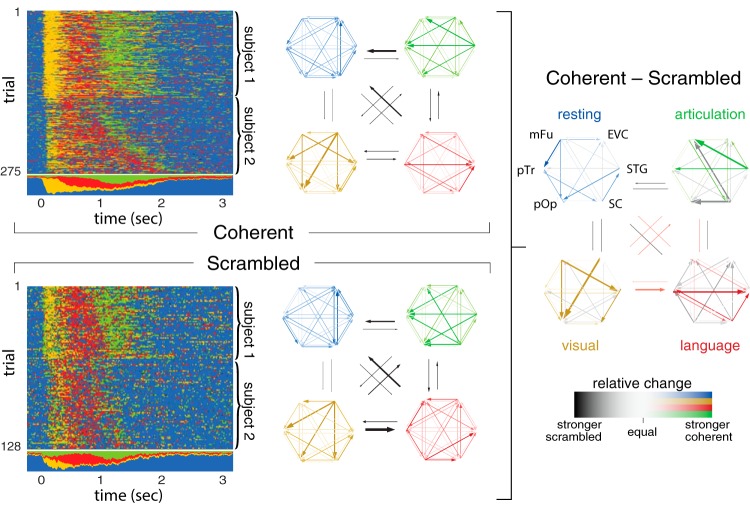
Multi-subject integration (anatomic grouping) of the two left-hemispheric recordings for coherent (top left) and scrambled (bottom left) stimulus condition. Differences in information flow between both stimulus conditions are shown on the right, where colored and gray arrows denote excess activity in the coherent and scrambled condition, respectively.

**Figure 9.**
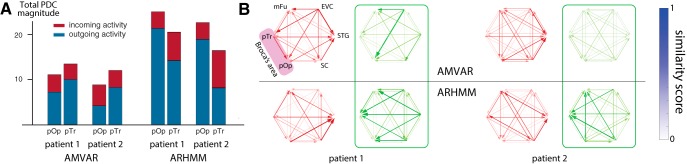
Comparison of AMVAR and ARHMM estimates of activity in Broca’s area during articulation. The ARHMM shows stronger total interactions than AMVAR analysis, especially for Broca’s area. ***A***, Total incoming and outgoing activity (red and blue, respectively) for Broca’s pars opercularis (pOp) and pars triangularis (pTr) during articulation, according to AMVAR and ARHMM models. ***B***, Connectivity graphs for AMVAR (top) and ARHMM (bottom), shown for two left-hemispheric patients for the language processing (red) and articulation state (green).

Using ARHMM inference in combination with Bayesian model selection (Fig. 5), we analyzed the network dynamics for each subject independently (Fig. 6). These analyses converged on models for all subjects featuring dynamics that depended on three time lags and were best explained by four network states.

Prestimulus and postarticulation activity patterns were predominantly assigned to one state, which we therefore named “resting.” Immediately following picture presentation, a second state dominated for ∼250 ms, which we named “visual processing.” A prominent feature of this network state was information flow from early visual cortex and middle fusiform gyrus. The next state is characterized by interactions distributed throughout the network, but most strongly driven by frontal regions. We named this state language processing. Comparing our inference results for both hemispheres, we find that the language processing state was only pronounced in the recordings of language-dominant cortex (Fig. 6), consistent with a left-lateralized language production network. During articulation, we observed a fourth state we named “articulation” which featured greater information flow from subcentral gyrus and superior temporal gyrus back to the language processing network. The ARHMM model reveals that neural interactions transition back to the resting state following each completed articulation.

To check whether multi-electrode activity patterns suggested different state sequences than implied by the best electrode in each region, we repeated our ARHMM analysis using a weighted average of electrodes in each region. The weighting was selected to extract the principal component, i.e., the multi-electrode pattern with the widest range of gamma-band power for each region. When fit to these patterns, the model produced higher state probabilities and exhibited lower similarity across subjects (Extended Data Fig. 6-1), but were otherwise consistent with the results from individually selected electrodes for each region.

An indication that the network states found by the ARHMM reflect task-relevant neural computations is that they predict the onset of articulation. The duration of the visual processing state was uninformative about this onset time (Fig. 7*A*), but both the duration (Fig. 7*B*) and termination (Fig. 7*D*) of the language processing state were significantly related to reaction time. This is consistent with variable difficulty in identifying word names for the heterogeneous stimuli. For one of the two patients with left hemisphere recordings, the duration of the articulation state was negatively related to articulation onset (Fig. 7*C*).

Across these three patients and across 120–160 trials, we find that the inferred network states follow a reliable state sequence (Fig. 6*A*). Moreover, each state’s interactions between brain regions were similar between patients, as measured by PDC (Fig. 6*B*). Our measurements do not cover the complete visual and language processing networks, and these areas are unlikely to have exclusively direct connections. Nonetheless, the analysis still indicates that interactions between early visual cortex and the language areas have directionality, even if mediated by some unmeasured areas.

The control condition of scrambled images still provides a visual stimulus and requires articulation, but does not bind a specific concept (aside from the general category of scrambled). We measured differences in network dynamics during coherent and scrambled image naming by inferring the network structure and states for each condition separately. To improve the ARHMM inference and to make it more robust with respect to subject-to-subject variability, we combined trials from both left-hemisphere data sets, effectively assuming that electrodes in both recordings were anatomically homologous.

Neural activity in both stimulus conditions were described by comparable networks, but exhibited specific differences in connectivity between the network nodes (Fig. 8, left). The differences were most pronounced in the active states: visual processing, language processing, and articulation. This suggests that the observed differences are due to condition-specific network activity.

The strongest network connections in each state were generally suppressed when viewing scrambled images. During the visual processing state for coherent images, early visual cortex and middle fusiform gyrus had stronger influences on frontal regions than for scrambled images. In the language processing state, the superior temporal gyrus received more input from subcentral gyrus and Broca’s area (pars triangularis and pars opercularis) when naming coherent images. The articulation state for coherent images shows increased information flow emanating from superior temporal gyrus, while the same state for scrambled images shows increased information flow from subcentral gyrus. This dissociation between auditory and sensorimotor cortex responses to coherent and scrambled images could be driven by learning effects from consistent repetition of the stereotyped response scrambled.

As described above, we find that Broca’s area, pars triangularis and pars opercularis, strongly interacts with the overall naming network both immediately following picture presentation and during articulation (Fig. 9). This is in contrast to Flinker et al. (2015), where trial-averaged interaction measures including AMVAR (Ding et al., 2000) revealed Broca’s area activity only before articulation onset, leading to the conclusion that this region exclusively supports articulatory planning and not articulatory execution. While AMVAR also finds no significant interactions for pars triangularis and opercularis during articulation, ARHMM reveals incoming information flow during articulation. This fits best with a feedback mechanism in which each network state terminates activity in the dominant nodes of the prior network state.

## Discussion

We identified meaningful cortical states at the single-trial level with a simple but powerful nonlinear model of neuronal interactions, the ARHMM, a dynamical Bayesian network. We used this model to interpret intracranial recordings at electrodes distributed across the language-dominant hemisphere during a classic language production paradigm, picture naming. The model revealed a consistent progression through three network states that were distinguished by their interaction patterns: visual processing, language processing, and articulation. This analysis of high-resolution intracranial data shows the ARHMM to be a useful tool for parsing network dynamics of human language, and more broadly for quantifying network dynamics during human cognitive function.

### Methodological benefits

We have demonstrated that Bayesian dynamical networks can extract structure of dynamical and distributed network interactions from ECoG data and reveal an interpretable sequence of network states unfolding during language processing. The ARHMM algorithm provides a principled statistical algorithm that can learn brain states in an efficient and unsupervised fashion. Some of the severe limitations of conventional windowed MVAR methods are alleviated by this kind of analysis. One particular virtue is that the ARHMM is sensitive to trial-by-trial timing variations; conventional methods that fail to account for this variability will dilute their estimates of network structure over time, lumping distinct states together or wrongly splitting them. The ARHMM is sensitive to the distributions of network activity, and this allows more refined inferences than the usual *k*-means clustering, which assumes all states have equal, isotropic variability. Furthermore, Bayesian inference of the ARHMM incorporates uncertainty, which can be used to determine whether any differences in brain states between subjects, groups, or conditions are significant. Some of these benefits have been observed when classifying epileptic activity (Baldassano et al., 2016), identifying processing stages encoded in task-dependent patterns of electrode activation (Borst and Anderson, 2013), or studying working memory in slow, high-dimensional fMRI signals (Taghia et al., 2018). Here we have demonstrated that these models are useful also for discriminating between cognitive states in normal brain function measured at the high temporal resolution provided by ECoG measurements. All of these advantages could benefit future work in mining and understanding ECoG data about intact brain computation.

### Sequence of states

This work reveals a clear and fairly consistent progression of neural dynamics through three active states in each picture naming trial, which we associate with visual processing, language processing, and finally articulation. These states appear to be meaningful because they are well-aligned with observable behavioral events: picture presentation or articulation. Visual processing was the dominant mode of neural activity for ∼250 ms after picture presentation; language processing followed until articulation onset; and articulatory execution lasted for the duration of overt speech production.

The serial progression through these three states is in striking contrast to fully interactive language models, which expect a relatively homogeneous temporal blend of all three states from picture presentation through the end of articulation. Our findings therefore suggest that these states correspond to discrete cognitive processes without strong temporal overlap or interaction (Indefrey and Levelt, 2004).

These cognitive processes, visual processing, language processing, and articulatory execution, are quite broadly defined. In particular, the language processes for speech production are thought to invoke separable cognitive processes supporting semantic, lexical, and phonological elements (Levelt, 1989). Similarly, articulation could be expected to invoke additional stages relating to phono-articulatory loops. The ARHMM did not identify additional distinct states within the language processing interval that might correspond to such elemental processes. If these elemental processes are highly interactive (Dell, 1986), then these elemental processes may effectively blend together into the single state in an ARHMM with recordings at a handful of distributed electrodes. Otherwise, more trials may make it easier to find evidence of such brief processes. The disambiguation of these additional intermediary states is a focus of ongoing work.

### Network interactions

ARHMMs are state-switching models driven by linear, pairwise, directional interactions between network nodes. Consequently, each state is defined by a unique interaction structure. We found that networks in left and right hemispheres had similar structures during visual processing and articulatory execution. Visual processing featured strong interactions between early visual cortex and the rest of the language network. In articulatory execution, interactions were strongest between perisylvian regions: pars triangularis, pars opercularis, subcentral gyrus, and superior temporal gyrus. Prearticulatory language processing showed a distributed set of interactions across ventral temporal and lateral frontal cortex limited to the language-dominant hemisphere. This evidence is consistent with a bilateral visual processing system (Salmelin et al., 1994) that converges for picture naming to a lateralized language network (Frost et al., 1999), which in turn drives a bilateral articulatory system (Hickok and Poeppel, 2007).

The contrast between interactions during coherent and scrambled naming trials revealed specific cognitive processes supported by discrete sub-networks. Coherent images induced stronger interactions from ventral temporal to lateral frontal regions during visual and language processing, as well as from superior temporal gyrus to the rest of the network during articulation. Scrambled images do not evoke a specific object representation in the brain, requiring only a stereotyped response (scrambled). These two functional distinctions between the coherent and scrambled conditions imply that language planning processes are subserved by temporal-to-frontal connections, while phonological motor processes are subserved by frontal-to-temporal connections (Whaley et al., 2016).

### Switching linear dynamics versus nonlinear dynamics

General nonlinear dynamical systems are too unconstrained to be a viable model for brain activity. Two approaches to constraining the nonlinear dynamics are to use a model that represents smooth nonlinear dependencies, or to use a switching model which combines simpler local representations. Our model is an example of the latter type. One could use hard switches between distinct models, as we and others do (Sahani, 1999b; Ghahramani and Hinton, 2000; Fox et al., 2009; Linderman et al., 2017); or one could use smooth interpolations between them (Wang et al., 2006; Yu et al., 2007). Each system will have computational advantages and disadvantages, and it would be beneficial to compare these methods in future work.

### Activity-dependent switching versus activity-independent switching, and recognition models versus generative models

Our method is based on a generative model, an assumed Bayesian network that is credited with generating our observed data. One feature of this generative model from Equations 2, 3 is latent brain states that transition independently of neural activity. This model therefore cannot generate context- or activity-dependent interactions. Furthermore, the assumed Markov structure enforces exponentially-distributed transitions between network states, which may not reflect the real dynamics of computations. Finally, some of the temporal dynamics of brain states should reflect the interactions’ explicit dependence on sensory input, which is neglected in our model.

There are two properties of our model that mitigate these concerns. First, if multiple latent states produce the same observations, then this could produce non-exponentially-distributed transitions between distinct observable states (Limnios and Oprisan, 2012). Second, even if the model itself corresponds to a prior distribution that does not quite have the desired properties, when fit to data the posterior distribution can nonetheless exhibit context-dependence and non-exponential transitions between latent states. Essentially, the model’s prior provides enough structure to eliminate many bad fits, while remaining flexible enough to accommodate relevant dynamic neural interactions.

There are many opportunities to generalize the ARHMM to accommodate more desired features. While one always must balance model complexity against data availability, which is typically highly limited for human patients, one can fruitfully gain statistical power by combining data from different subjects to create models with some common behaviors and some individual differences. When applied to common tasks, such models could automatically identify universal properties of neural processing across subjects and even across different tasks.

## Synthesis

Reviewing Editor: Tatyana Sharpee, The Salk Institute for Biological Studies Decisions are customarily a result of the Reviewing Editor and the peer reviewers coming together and discussing their recommendations until a consensus is reached. When revisions are invited, a fact-based synthesis statement explaining their decision and outlining what is needed to prepare a revision will be listed below. The following reviewer(s) agreed to reveal their identity: Avniel Ghuman.

The authors use autoregressive hidden Markov models (ARHMM's) to analyze ECoG data from 3 human subjects during a speech production task. The inferred states and their corresponding linear dynamics offer a network interpretation of interactions between six brain regions. Models like these offer a simple yet powerful way to go beyond simple linear dynamics while still retaining interpretable structure in the form of discrete states. The reviewers raised a few concerns that should be addressed prior to publication.

Reviewer 1:

This work combines ECoG recordings from 3 patients with ARHMMs to examine the neurodynamics of picture naming. The methods are rigorous and the results interesting. In particular, this introduces a methodology that could have broad implications and applications for invasive and non-invasive electrophysiology. Furthermore, the results, while somewhat preliminary given the small patient number, are potentially important for understanding picture naming.

Overall this is an interesting study that applies rigorous and innovative methodology. Some things should be clarified and adjusted.

Some comments

1. It is not accurate to call the high frequency broadband signal “high frequency gamma oscillations” as this is a broadband signal (60-120 Hz in this case) and is unlikely to be oscillatory. While the terminology has not been settled, this is often termed “high frequency broadband” or “broadband gamma” rather than “Gamma-band activity” and all references to “oscillations” should be removed unless the authors can definitively demonstrate that their results relate to a narrow band of the spectrum.

2. How were 60-120 Hz bandpass filtered data downsampled to 200 Hz? In general, downsampled frequencies should be 2-3 times the highest frequency of the bandpass.

3. How were the electrodes “with significant activity from each region” chosen?

4. It would be useful to know if the end point of the semantic state correlates with articulation onset.

5. The use of HMMs for BCI should at least briefly be discussed as they have been used extensively there, including with ECoG.

6. The use of HMMs in neuroscience to assess the neural basis of cognitive models more broadly should be briefly discussed. For example, John Anderson has used HMMs in conjunction with MEG and EEG data to examine the neural basis of ACT-R models. While autoregressive methods aren't used in those cases, some of the neural signal estimation procedures have similar effects to an autoregressive model. This previous work should be discussed in section 5.1 .

7. It would be good to model an SNR in between 0.3 and 3 in figure 3 as many neural measurements fall somewhere in between those values.

8. The “visual state” in the blue patient in figure 6A is rather long. This should be noted.

9. In the abstract, it should be made clear that the names of the states are hypotheses and supported by the data, but not a direct inference from results themselves.

Reviewer 2:

1. Why apply the model to high frequency gamma oscillations rather than directly to the ECoG measurements? Please make this clear in the paper and discuss possible issues with bandpass filtering the raw signal and then performing frequency-based analyses (PDC) of the inferred dynamics parameters.

2. The terms “switching linear dynamical system” and “autoregressive hidden Markov model” refer to two separate models in the machine learning community (c.f. Murphy (2012) “Machine Learning: A Probabilistic Perspective”). Please be more precise with nomenclature on page 3, section 5.4, etc.

3. By only using one electrode per brain region per individual (Sec 3.5) you must be discarding a huge amount of data. (Sec 3.4 says there are 615 electrodes in total.) How do the results change if you use

all electrodes? What if you perform dimensionality reduction (e.g. PCA) separately for each brain region instead of choosing only one electrode? How sensitive are the results to choice of electrode? Please include analyses of this form, even if they are supplementary material.

4. The ARHMM specified in Eq (1) does not include an affine term. Including a bias can have significant effects on the inferred states, even if the data is mean-centered. How do your results change when you include a state-dependent bias b_{z_t} in the model? Also, your formulation of the autoregressive dependencies differs slightly from other treatments in using A_{z_{t-\tau}} instead of A^{(\tau)}_{z_t};

as written, x_t depends on (z_{t-N_\tau}, ... z_t), which is not consistent with the graphical model shown in Fig 2A.

5. How do you set the “stickiness” parameter u in Eq (4)?

6. The PDC looks (a bit) like a Fourier transform of the discrete-time AR dynamics for a given state and “edge” of the network. When you integrate the PDC over all frequencies, as described in Sec 3.7, are

you effectively performing something like an inverse Fourier transform? Please discuss the interpretation of the integrated PDC.

7. Figure 3D has many blank panels. Please fix. (If the correlations are effectively zero then say so in text and omit the blank panels.) Also, Figure 3 caption overlaps the page number.

8. Please show the BIC curves for choosing the number of states and lags and compare to held-out log likelihood (marginalizing out the discrete states). In many real world analyses, the held-out log likelihood continues to increase as states are added. Does that happen here as well, but not enough to overcome the BIC complexity penalty?

9. The text says the inferred networks are similar across patients, but the network comparisons for the ARHMM in Fig 8B look very different. Please explain and quantify the similarities. How are you fitting the model for multiple patients? Are they fit simultaneously or fit separately and the aligned post-hoc with, e.g., the Hungarian algorithm?

## Author Response

Authors' Replies to Reviewers

Synthesis Statement for Author (Required):

The authors use autoregressive hidden Markov models (ARHMM's) to analyze ECoG data from 3 human subjects during a speech production task. The inferred states and their corresponding linear dynamics offer a network interpretation of interactions between six brain regions. Models like these offer a simple yet powerful way to go beyond simple linear dynamics while still retaining interpretable structure in the form of discrete states. The reviewers raised a few concerns that should be addressed prior to publication.

We appreciate the reviewers' careful reading and thoughtful comments. We have implemented their suggestions, which have provided useful new results and improved the clarity of the manuscript. Below and in the revised manuscript we have noted textual changes using >>. Equations that cannot be reproduced here directly are written in TeX format.

Reviewer 1:

This work combines ECoG recordings from 3 patients with ARHMMs to examine the neurodynamics of picture naming. The methods are rigorous and the results interesting. In particular, this introduces a methodology that could have broad implications and applications for invasive and non-invasive electrophysiology. Furthermore, the results, while somewhat preliminary given the small patient number, are potentially important for understanding picture naming.

Overall this is an interesting study that applies rigorous and innovative methodology. Some things should be clarified and adjusted.

Some comments

1. It is not accurate to call the high frequency broadband signal “high frequency gamma oscillations” as this is a broadband signal (60-120 Hz in this case) and is unlikely to be oscillatory. While the terminology has not been settled, this is often termed “high frequency broadband” or “broadband gamma” rather than “Gamma-band activity” and all references to “oscillations” should be removed unless the authors can definitively demonstrate that their results relate to a narrow band of the spectrum.

We thank the reviewer for this clarification and have removed all references to gamma oscillations, replacing it with “broadband gamma power”.

2. How were 60-120 Hz bandpass filtered data downsampled to 200 Hz? In general, downsampled frequencies should be 2-3 times the highest frequency of the bandpass.

We are analyzing the broadband gamma power (i.e. the envelope of the raw trace) which does not change faster than half the final downsampled sampling rate (100 Hz). The data were first filtered and Hilbert transformed to obtain the bandpass analytic signal. From this, instantaneous power was extracted. This resulting power trace was then downsampled. In this manner, we do not violate the Nyquist rate. We have revised the description of this procedure in the methods to be more explicit:

>> “The analytic signal was generated with frequency domain bandpass Hilbert filters featuring paired sigmoid flanks (half-width 1 Hz) (Forseth et al., 2018). Instantaneous power was subsequently extracted from the analytic signal, normalized by a pre-stimulus baseline level (700 ms to 200 ms before picture presentation), and then downsampled to 200 Hz” (page 8).

3. How were the electrodes “with significant activity from each region” chosen?

In regions with several candidate electrodes, we always used the one with the greatest total broadband gamma power increase from baseline (constrained to the period between picture presentation and the completion of articulation). We state this in the methods section. Please note that an alternate approach suggested by Reviewer 2, namely to combine all electrodes within a region using PCA to create a pooled “superelectrode”, did not alter our results or conclusions.

>> “In each individual, we selected the most active electrodes from each region (Figure 1A,B). In a separate analysis to combine information from multiple sources, we grouped electrodes from each brain region, computed the principal components of the high gamma-band power across electrodes within each region, and selected the leading component as a meta-electrode to represent each region.” (page 8)

4. It would be useful to know if the end point of the semantic state correlates with articulation onset.

We appreciate this suggestion from the reviewer. We identify the end of the ‘language processing state’ as the last time between picture presentation and articulation completion when the majority of states within a sliding 100 ms window were identified as ‘language processing’. The end of this state does indeed correlate strongly with the onset of articulation. This has been added as Figure 7D. We describe our method to measure state times and durations in the methods section:

>> “To estimate the cumulative durations of each state, we first filter the states by computing the most probable state within a sliding 200 ms time window. We then integrate the total time within these filtered state sequences when each state dominated, from stimulus onset to 3200 ms after stimulus onset. We identify the end of the ‘language processing state’ as the last time between picture presentation and articulation completion when the majority of states within a sliding 100 ms window from stimulus onset to articulation offset were identified as ‘language processing’.” (page 15)

5. The use of HMMs for BCI should at least briefly be discussed as they have been used extensively there, including with ECoG.

See answer to 6:

6. The use of HMMs in neuroscience to assess the neural basis of cognitive models more broadly should be briefly discussed. For example, John Anderson has used HMMs in conjunction with MEG and EEG data to examine the neural basis of ACT-R models. While autoregressive methods aren't used in those cases, some of the neural signal estimation procedures have similar effects to an autoregressive model. This previous work should be discussed in section 5.1.

We agree with the reviewer that these would be good additions to the paper, so we have added a brief description of this and related work in the introduction and discussion, and highlighted the advances uniquely contributed by our work:

>> “Hidden Markov Models have become a useful tool in characterizing brain dynamics on a multitude of spatial and temporal scales. They have proved valuable in brain-computer interfaces (Al-Ani and Trad, 2010; Kemere et al., 2008), modeling the sequential structure of cognitive processes using functional magnetic resonance imaging (Anderson, 2011), and or describing brain states and behavioral states Abeles et al. (1995); Sahani (1999a); Escola et al. (2011).” (page 3)

“Some of these benefits have been observed when classifying epileptic activity (Baldassano et al., 2016), identifying processing stages encoded in task-dependent patterns of electrode activation (Borst and Anderson, 2013), or for studying working memory in slow, high-dimensional fMRI signals (Taghia et al., 2018). Here we have demonstrated that these models are useful also for discriminating between cognitive states during normal brain function measured at the high temporal resolution provided by ECoG measurements.” (page 26)

7. It would be good to model an SNR in between 0.3 and 3 in figure 3 as many neural measurements fall somewhere in between those values.

We agree, and have updated Figure 3 accordingly with a range of SNRs: 0.3, 1.5, 3, and 30. (page 14)

8. The “visual state” in the blue patient in figure 6A is rather long. This should be noted.

We noticed that there are spurious fluctuations of the visual state occurring throughout all trials, irrespective of time. These uncorrelated fluctuations are present during both active, and even inactive states (resting state). Our previous algorithm counted these fluctuations as true state occupancies, not as artifacts. Since the results (Viterbi trace) for patient 1 show more of fluctuations for patient 1 than for patient 2, counting state occupancies directly (without smoothing) introduces a bias of longer visual state durations for patient 1, as correctly pointed out by the reviewer. By taking a moving average (instead direct counts), we are able to remove these random fluctuations, thus removing the bias of longer visual state durations for subject 1.

9. In the abstract, it should be made clear that the names of the states are hypotheses and supported by the data, but not a direct inference from results themselves.

This is a good suggestion to prevent reader confusion. We have adjusted the abstract text accordingly:

>> “the data support the hypothesis of a fixed-length visual processing state, followed by a variable-length language state, and then by a terminal articulation state.” (page 1)

Reviewer 2:

1. Why apply the model to high frequency gamma oscillations rather than directly to the ECoG measurements? Please make this clear in the paper and discuss possible issues with bandpass filtering the raw signal and then performing frequency-based analyses (PDC) of the inferred dynamics parameters.

In principle our method applies to a wide variety of signals, including the raw waveform itself, power at other frequency bands, and multidimensional power at different frequencies. We focus on broadband gamma power because this measure has been shown to especially relevant to the study of cognition (Crone et al., 2001; Jacobs and Kahana, 2010; Lachaux et al., 2012). Broadband gamma is thought to arise from the focal summation of post-synaptic currents coupled with a surge in spike rate (Lachaux et al., 2012; Manning et al., 2009), indexing local processing (Cardin et al., 2009; Logothetis, 2003; Magri et al., 2012). It correlates strongly with the fMRI blood-oxygen-level dependent (BOLD) signal and, with its superior temporal resolution, can precisely characterize inter-regional timing (Conner et al., 2011; Mukamel et al., 2005). We have added motivation for this focus in the introduction:

>> “We concentrate our analysis on broadband gamma power, a neural measure that is thought to arise from spike surges, and serves as a measure of local cognitive processing (Logothetis2003, Lachaux2012).” (page 4)

Note that our analysis extracts structure present in co-fluctuations of the broadband gamma power across electrodes. This power envelope necessarily changes more slowly than the underlying waveform, as mentioned above. As such, a frequency-based analysis on this envelope is distinct from the bandpass properties of the carrier.

2. The terms “switching linear dynamical system” and “autoregressive hidden Markov model” refer to two separate models in the machine learning community (c.f. Murphy (2012) “Machine Learning: A Probabilistic Perspective”). Please be more precise with nomenclature on page 3, section 5.4, etc.

We have updated our terminology accordingly.

3. By only using one electrode per brain region per individual (Sec 3.5) you must be discarding a huge amount of data. (Sec 34 says there are 615 electrodes in total.) How do the results change if you use all electrodes? What if you perform dimensionality reduction (e.g. PCA) separately for each brain region instead of choosing only one electrode? How sensitive are the results to choice of electrode? Please include analyses of this form, even if they are supplementary material.

We have implemented the reviewer's suggestion to perform dimensionality reduction (PCA) for each brain region. This analysis is now included as Extended Data in support of the individual electrode results in Fig. 6.

>> “In a separate analysis to combine information from multiple sources, we grouped electrodes from each brain region, computed the principal components of the high gamma-band power across electrodes within each region, and selected the leading component as a meta-electrode for each region.” (page 8)

>> “To check whether multi-electrode activity patterns suggested different state sequences than implied by the best electrode in each region, we repeated our ARHMM analysis using a weighted average of electrodes in each region. The weighting was selected to extract the principal component, i.e. the multi-electrode pattern with the widest range of gamma-band power for each region. When fit to these patterns, the model produced higher state probabilities (Extended Data Fig. 6-1) and exhibited lower similarity across subjects, but were otherwise consistent with the results from individually selected electrodes for each region.” (page 20)

4. The ARHMM specified in Eq (1) does not include an affine term. Including a bias can have significant effects on the inferred states, even if the data is mean-centered. How do your results change when you include a state-dependent bias b_{z_t} in the model? Also, your formulation of the autoregressive dependencies differs slightly from other treatments in using A_{z_{t-\tau}} instead of A^{(\tau)}_{z_t}; as written, x_t depends on (z_{t-N_\tau}, ... z_t), which is not consistent with the graphical model shown in Fig 2A.

We are thankful that the reviewer spotted this missing affine term. We did include it in the original model (and the code accompanying our submission), but did not describe it in the methods. We have corrected this omission and detailed its initialization (page 19). Additionally, we have updated our autoregressive dependency graph (Figure 2A) and Equation 3 to match our mathematical formulation.

5. How do you set the “stickiness” parameter u in Eq (4)?

The stickiness parameter was a hyperparameter set to u=0.5 (this information has been added to the Methods section, page 12) to reduce spurious state fluctuations while maintaining sensitivity to state changes. Our results were insensitive to this choice within a wide range of values.

6. The PDC looks (a bit) like a Fourier transform of the discrete-time AR dynamics for a given state and “edge” of the network. When you integrate the PDC over all frequencies, as described in Sec 3.7, are you effectively performing something like an inverse Fourier transform? Please discuss the interpretation of the integrated PDC.

Yes, the integrated PDC magnitude (word added in revision) is the zero-lag term of the inverse Fourier transform: $\sum_f |\pi|_{zjk}(f)=\sum_f|\pi|_{zjk}(f)e^{if0}=|\tilde{\pi}|_{zjk}(t=0)$. Without the magnitude or the normalization, this would retrieve the autoregressive coefficient at zero lag, which is zero by construction. However, the magnitude and normalization change the picture. Since the PDC normalization emphasizes the outgoing signals from node k at frequency f, the integrated PDC magnitude reflects the total outbound strength to a given target, at all frequencies.

7. Figure 3D has many blank panels. Please fix. (If the correlations are effectively zero then say so in text and omit the blank panels.) Also, Figure 3 caption overlaps the page number.

These panels were not blank, but actually showed that residuals were quite low. However, since these low residuals were not visually informative, we have removed those panels and stated so in the text:

>> “For the SNRs tested, the residual temporal correlations between data and model fit are small, indicating that the model is reasonable even for low SNR.” (page 15)

8. Please show the BIC curves for choosing the number of states and lags and compare to held-out log likelihood (marginalizing out the discrete states). In many real world analyses, the held-out log likelihood continues to increase as states are added. Does that happen here as well, but not enough to overcome the BIC complexity penalty?

We added a model comparison showing the BIC and held-out likelihood in Fig. 5. As mentioned by the reviewer, the likelihood continues to increase as states are added, neutralizing the BIC complexity penalty. Using a minimum threshold for the decrease in BIC, we find 4 states and AR order of 3 to be optimal, as mentioned in the Results (page 19).

9. The text says the inferred networks are similar across patients, but the network comparisons for the ARHMM in Fig 8B look very different. Please explain and quantify the similarities. How are you fitting the model for multiple patients? Are they fit simultaneously or fit separately and the aligned post-hoc with, e.g., the Hungarian algorithm?

Models were fit to each patient separately. We define the dissimilarity between two matrices, A and B, through the Frobenius norm of their difference (Eq. 7), as described in the methods section:

>> “In order to compare the states across subjects, we define a dissimilarity score based on a distance, d(A,B), between two matrices of integrated PDC magnitudes:

$$d(A,B) = \| \frac{A}{\|A\|_F} - \frac{B}{\|B\|_F}\|$$

where $\|A\|_F=\sqrt{\sum_{ij}|A_{ij}|^2}}$ denotes the Frobenius norm. We calculate this difference for all pairs of states for different subjects.” (page 16)

We have now added panel C to Figure 6 to quantify these dissimilarities between all pairs of states, both within a subject and across subjects. Although network state structures differ across subjects, corresponding states do have the closest match.
